# Preference of Older Adults for Flexibility in Service and Providers in Community-Based Social Care: A Discrete Choice Experiment

**DOI:** 10.3390/ijerph19020686

**Published:** 2022-01-08

**Authors:** Kailu Wang, Eliza Lai-Yi Wong, Amy Yuen-Kwan Wong, Annie Wai-Ling Cheung, Eng-Kiong Yeoh

**Affiliations:** Centre for Health Systems and Policy Research, JC School of Public Health and Primary Care, Faculty of Medicine, The Chinese University of Hong Kong, Hong Kong, China; kailuwang@cuhk.edu.hk (K.W.); amy.wong@cuhk.edu.hk (A.Y.-K.W.); anniewlcheung@cuhk.edu.hk (A.W.-L.C.); yeoh_ek@cuhk.edu.hk (E.-K.Y.)

**Keywords:** long-term care, home and community-based care, self-directed care, consumer-directed care, personal budgets, decision-making, conjoint analysis, willingness to pay

## Abstract

Empowerment of control and choice of the service users in health and social care has been incorporated into service provision in various countries. This study aimed to elicit the preference of community-based long-term care (LTC) service users on levels of flexibility in service provision. A discrete choice experiment was performed among older community care service users to measure their preference for attributes of LTC services identified from a prior qualitative study. Each participant was asked to make choices in six choice tasks with two alternatives of hypothetical LTC services that were generated from the attributes. A generalized multinomial logistic model was applied to determine the relative importance and willingness to pay for the attributes. It found that the participants preferred multiple flexible providers, determining services by themselves, meeting case managers every month and social workers as sources of information on service provision. Significant preference heterogeneity was found for flexibility in providers and flexibility in services between those with and without activity of daily living impairment. The findings highlighted the preference of older adults for greater flexibility in LTC, while they rely heavily on social workers in decision making. The enhancement of flexibility in LTC should be supported by policies that allow the older service users to make decisions based on their own preferences or communication with social workers instead of determining the services and providers for them. Options should be offered to users to decide their preferred level of flexibility to better reflect their divided preferences.

## 1. Introduction

The “money-following-users” approach, which has been implemented in the public-funded long-term care (LTC) system of Hong Kong, China, since 2013 [1,2], aims to enable service users to gain greater flexibility over their own care arrangement and enable them to stay at home and in the community they are familiar with (i.e., ageing in place) by providing benefits in the form of a voucher, i.e., a community care service voucher (CCSV) for older adults to purchase home- and community-based LTC services [2,3]. In Hong Kong, the older person’s eligibility for using public-funded LTC services needs to be ascertained by a standardized care needs assessment mechanism for elderly service (SCNAMES) based on their physical and mental functions as well as available social support and living environment. The users eligible for public-funded LTC service are allowed to choose between traditional in-kind services with determined service providers and service packages and the pilot CCSV scheme adopting novel “money-following-users” approach. This approach shares a similar concept with “self-directed care” or “consumer-directed care” implemented in several countries in the Organization for Economic Co-operation and Development (OECD), including the United States (e.g., Cash and Counseling Demonstration and Evaluation), Australia (e.g., Consumer Directed Care) and some European countries (e.g., Personal Health Budget in UK, “Persoonsgebonden budget (PGB)” in the Netherlands), which also aim to empower LTC service users and grant them more control over their daily services by offering personal care budgets to them and allowing them to make choices about their care service and service delivery, particularly about home- and community-based services [4,5,6,7]. Self-directed or consumer-directed care usually serves as a tool and mechanism in facilitating the exercise of autonomy, which was found to be essential to the quality of life of older persons and enabled them to make meaningful choices in LTC that meet their unique needs [8]. It was also found to lower the unmet needs for home-based healthcare and transportation services in the US [9].

As the service users play an essential role in service delivery under the “money-following-users” approach because of the empowerment of control and choice over the LTC services, their preferences need to be considered in service provision to better meet their demands. LTC programmes have various designs in financing, organization, delivery and regulation across countries and regions, which leads to a wide spectrum ranging from greater flexibility for individual service users to more involvement of the authorities [4,6,10]. However, previous local studies of this approach failed to provide adequate information on the preference of older adults for gaining greater flexibility in LTC services [1,2,3,11]. Internationally, the complexity of design of self-directed/consumer-directed care programmes in different countries and regions as well as variety in socio-demographical background of policy implementation make it difficult to make comparisons among the programmes to find out whether greater flexibility contributes to a higher preference for the services [6]. There were a limited number of published studies showing the preference of social care services for flexibility, control and choice in the services. A systematic review on international self-directed/consumer-directed care programmes also indicated that it was unclear what factors in the design of self-directed care matter to older service users [12]. Thus, there is a need to quantify the level of preference for greater flexibility in LTC services using willingness-to-pay (WTP) measurements, which can be used to inform the design and implementation of relevant services schemes, including the fee schedule and co-payment amount.

To meet the needs for different levels of flexibility, an important component in the “money-following-users” approach is support and assistance in the user’s capacity of decision making, which include the ability to obtain, understand and make adequate use of the information to support the choices made by the users [8,13,14]. The older LTC service user, who might suffer from physical or cognitive impairment, may have an inadequate capacity to make optimal decisions that fulfil their needs, which would eventually affect their level of flexibility as well as the care outcomes. To avoid this issue, consumer-directed or self-directed care service programmes in OECD countries often provide case management or counselling services as support or a safeguard for decision making for the users to assist them in making a meaningful decisions [4]. In light of this, the preference for such support in decision making should also be measured to inform the design and pricing of these supports and assistances in LTC service provision and to compare it with the preference for greater flexibility to determine the relative importance among these characteristics.

To find out the preference for greater flexibility in choice, Kaambwa et al. [15] investigated the preference of older enrolees in Consumer Directed Care in Australia, which showed that being able to save unused funds for future use, flexibility in choosing service workers and flexibility in changing activities in care plans were found to be important to the care recipients, and these recipients preferred full or certain levels of flexibility in these aspects. It highlighted the advantage of using a discrete choice experiment (DCE) in policy research that allows the assessment of preference for policy design or characteristics not yet available in current practice. DCE can be performed not only among users of designated service programmes but also among potential users who have not received such services yet, which is useful for the planning and refinement of LTC policies and intervention design in advance of their implementation or extension to a wider range of users.

Considering all these factors, it is important to find out which attributes indicating flexibility in choice and support in decision making are essential and need to be considered in the design of “money-following-users” based on the preferences of older people. Based on the research question and a previous study on consumer-directed care [13], choice, information and capacity are three components that facilitate the delivery of the services. Choice refers to the adequacy of options in LTC and the flexibility to switch among them, while information and capacity refer to the availability of relevant information and the ability to make use of the information in making meaningful choices, respectively. Therefore, this study aimed to elicit the preference of older LTC services users in terms of level of flexibility and support in obtaining relevant information and in decision making capacity in a context of community-based LTC in Hong Kong with a DCE for refinement of the design and implementation of LTC services adopting “money-following-users”/self-directed care approach. It was hypothesized that the users preferred greater flexibility in choice in LTC services as well as greater support in decision making, including a higher frequency of case management and trustworthy information sources. The findings of this study can be useful to inform the design and implementation of the “money-following-users”/self-directed care approach in LTC service provision.

## 2. Materials and Methods

A cross-sectional survey incorporated with a DCE was conducted among older service users in Hong Kong during August and November 2018. This study was approved by the appropriate ethic committee. Written consent was obtained from all participants.

### 2.1. Study Sample and Data Collection

Since “money-following-users” is still in pilot phase in Hong Kong, this study targeted community-dwelling older persons who were users of a community-based LTC service, who represented a general older population in need of home- and community-based service and potential users of the CCSV. The sample of the survey consists of Cantonese speakers aged 65 and above who have used community care services of community centres for older adults (i.e., those who were registered as a member of local community centres, which is required by centres providing community-based services). The survey also excluded those with severe dementia, mental retardation or cognitive impairment as it was difficult for them to fully participate without the correspondingly support and assistance to give the needed responses to the questionnaire. Older adults living in hospital or residential care homes were also excluded.

Data collection was conducted in both in a household survey and survey in community centres to improve the representativeness of the study sample. The former one represents those who either had severer functional problems or lower willingness to participate in such activities, and the latter one represents those who usually participate in the activities of the centres and have more experience in community-based service and activities. As recommended by local social workers, the proportion of participants in the household survey should be between 1/3 and 1/2 of the sample to match the population proportion. For the household survey, rosters of the older adults receiving services from the community centres with their telephone numbers were provided by the centres. The older adults who gave initial consent for participation on the phone were interviewed at their home on a later date. For the community survey, older adults who joined the monthly meeting at the community centres were approached by the social workers during the meeting for the survey. The older adults who agreed to participate in the survey were interviewed in the centre on the reserved date. The participants were included from 3 community centres in Hong Kong. In both the survey and community centres, written informed consent was obtained, and the survey was conducted on a face-to-face basis.

### 2.2. Attributes and Experimental Design

Attributes and levels used in this study were derived from findings in a focus group discussion (N = 25) with stakeholders of social care services including older users of community service users, informal caregivers of community-dwelling older persons and staff working in social care, which aimed to identify the characteristics of “money-following-users” that are important for the older persons to decide whether to join a relevant scheme or not using a semi-structural discussion guide. With thematic analysis, the key attributes of “money-following-users” were found to be flexibility in service providers, flexibility in care services, case management level (in terms of meeting frequency), a source of information to support decision making, and expenses for the services (i.e., out-of-pocket monthly payment for the services) (Table 1). Flexibility in providers and services reflects the level of flexibility available to the users, while case management and a valid information source are often considered to be important for support in decision making, particularly for those with diminishing decision making capacity due to mental illness [16]. The values of out-of-pocket payment were set based on the community care service voucher pilot scheme phase II [17].

With the five attributes, a utility-neutral (i.e., zero prior mean, all levels of each attribute were set to be equally preferable in the design) D-optimal design was used to select 36 choice sets with 2 alternatives in each of the choice sets from a full factorial design that consists of 23,220 different choice sets [18]. These selected choice sets were allocated to six blocks in order to reduce the number of choice sets for each respondent to reduce cognitive burden. The respondents were randomly assigned to one of the six blocks and only needed to respond to six choice sets with two alternatives ach describing the different combinations of level of flexibility and support in decision making (i.e., the five attributes). The order of the choice sets was randomized. Multiple choice questions that asked the preference for each of the attributes were used as exercise tasks for respondents to become familiar with the attributes. A pilot survey of the DCE choice sets was conducted among 10 older persons for refinement (please see supplementary file).

### 2.3. Measurements

The questionnaire investigated the preference for the attributes of “money-following-users” using DCE and collected (1) the socio-demographic information of the subjects, including demographics, income, living arrangement, informal caregiver status, and (2) information on their functions and health conditions, including whether they were diagnosed with any chronic diseases, the utilization of the healthcare services, the Barthel activities of daily living (ADL) and the Lawton instrumental activities of daily living (IADL). ADL included 10 items, namely feeding, bathing, grooming, dressing, bowels, bladder, toilet use, transfers, mobility and stairs. IADL included 8 items, namely shopping, mode of transportation, meal preparation, housekeeping, laundry, phone calls, medication and managing finance (please see supplementary file).

### 2.4. Statistical Analysis

Based on the random utility theory, the utility of choosing alternative i (Ui) can be specified as below:

Ui = β0 + β1 ⋅ PROVIDERmultiple + β2 ⋅ SERVICEself +             β3 ⋅ SERVICEsocialworker + β4 ⋅ CASEMANAGEMENT every 3 months +                β5 ⋅ CASEMANAGEMENTevery 6 months + β6 ⋅ INFORMATIONsocialworker +      β7 ⋅ INFORMATIONfamily/friends + β8 ⋅ PAYMENT + εi(1)

As the utility cannot be measured directly, the choice of corresponding alternatives (1 = chosen, 0 = not chosen) was a dependent variable in the regression, and the attributes were independent variables. Generalized multinomial logistic model (GMNL) was adopted for analysis. Heterogeneity of the WTP across individuals needed to be accounted for in analysis to derive a more accurate WTP value. Under a few circumstances, there are substantial variations in preferences (i.e., WTP) for the attributes of a good or services across individuals, which are called preference heterogeneity [19]. In other circumstances, some preferences (i.e., WTP) of respondents with certain characteristics are more random than others, which are called scale heterogeneity [20]. GMNL is able to take into accounts both preference heterogeneity and scale heterogeneity by assuming the coefficient of each attribute levels is different across individuals (preference heterogeneity) and allows unequal error variances across individuals (scale heterogeneity) using random effect [21].

Interactions between the DCE attributes and socio-economic and health-related characteristics were also conducted to explore the differences in preferences among different populations using mixed multinomial logistic model (MIXL) to account for preference heterogeneity, as the GMNL could not provide converge in a few subgroups [22]. Sensitivity analysis was also performed using a latent-class logistic model which is reported in supplementary material, which assumes that the respondents can be assigned to different “latent classes” based on their preference patterns and estimates the preferences for these latent classes and their association with individual-level socio-economic and health-related characteristics (Tables S1 and S2, Supplementary file). Subgroup analysis was performed to identify the preference heterogeneity across individuals with different characteristics (Table S3, supplementary file).

The WTP can be calculated as the ratio of the coefficient of any of the attributes in the interests and the monetary term (i.e., monthly out-of-pocket payment) estimated by the GMNL regression model as above [23,24] using the below equation. The calculation of the confidence interval of WTP adopted the delta method.

(2) WTP=βkβ8

## 3. Results

### 3.1. Sample Characteristics

Of 326 older adults who agreed to join the survey, 318 of them finished the DCE part of the questionnaire, while the others did not complete the questionnaire due to the cognitive burden of DCE. The response rate was around 30% (around 1000 were invited for survey). Among them, 190 participants (59.7%) were interviewed in the community centres, while the other 128 (40.3%) were interviewed in their home. Of them, 76.4% were female, and the average age of the participants was 77.3 years (SD = 7.1 years). A total of 41.4% of them were living alone, and 35.6% of them were living with their spouse only. Around one-third of participants (34.0%) were living in public housing, and 28.9% of them reported a monthly income higher than HKD 5000 (USD 1 = HKD 7.8). Regarding their health status, 51.9% of them had IADL impairment, and 29.3% of them had ADL impairment (Table 2).

### 3.2. Preference for Level of Flexibility

The DCE results from the GMNL model are shown in Table 3. From the estimates of coefficients and WTP, the participants preferred multiple flexible providers and were willing to pay an extra monthly fee of USD22.2 (95%CI: USD13.7–30.6; USD1 = HKD 7.8) for this. They were also willing to pay USD29.0 (95%CI: USD19.2–38.8) for choosing the services by themselves or USD22.6 (95%CI: USD14.0–31.0) for social workers to choose based on their needs, USD12.6 (95%CI: USD2.6–22.4) for meeting a social worker every month for case management and USD14.7 (95%CI: USD5.6–23.7) for receiving information from social workers or the staff of care facilities rather than getting it from family members.

Following the WTP estimation for each attribute, the 10 combinations of attribute levels with the highest predicted WTP values are shown in Table 4. The WTP for them was calculated by summing up the WTP of each attribute with reference to the least-preferred combinations of attributes and levels (i.e., single fixed provider, fixed pre-determined service packages, meeting social workers every 6 months and getting information from family members or friends). The combination with the highest WTP was having multiple flexible providers, choosing the services by themselves, meeting with case manager every month and getting information from social workers. The respondents were willing to pay HKD 611/USD 78.4 monthly to switch from the least-preferred combination to this one.

### 3.3. Unobserved Preference Heterogeneity for Attribute Levels across Individuals

Unobserved preference heterogeneity refers to heterogeneity whose relationship with population characteristics is unknown, while observed preference heterogeneity means such a relationship has been determined in the analysis. The standard deviation of the variables in the model shown in Table 3 showed substantial preference heterogeneity found in flexibility in service provider and information source. Based on the coefficients of mean and the coefficients of standard deviation, around 66% of the participants preferred flexible providers, while 33% preferred single fixed provider. For information source, excluding those preferring getting information from family and friends, there were around 67% of the remaining respondents preferring social workers as an information source and 33% preferring receiving the information from the experience of a trial period (i.e., willing to pay more for a trial period of care services). These percentage estimations were calculated based on the formula Φ (−β mean/βsd), where Φ(k) refers to a cumulative distribution function calculating the area under the standard normal distribution from −∞ to k [25].

### 3.4. Observed Preference Heterogeneity across ADL and Income Subgroups

In order to find out the preference heterogeneity across subgroups with different socio-economic and health-related characteristics, interactions between these characteristics and the attributes were tested in the regression model. The interactions with *p* ≥ 0.05 were excluded from the model. In the end, it was found that the interactions between ADL impairment and flexibility in providers and the interaction between ADL impairment and flexibility in services were significant (*p* < 0.05) (Table 5). The older persons with ADL impairment preferred multiple flexible providers or greater flexibility in providers, while they preferred services chosen by oneself and greater flexibility in care services less.

A latent class model was used as a supplementary analysis for exploring the preference heterogeneity (supplementary file, Tables S1 and S2). It was found that older persons with a higher income are less sensitive to out-of-pocket payment (i.e., lower absolute value of coefficient β compared with other latent classes). The older persons with ADL impairment are more likely to have greater preference for greater flexibility in providers (i.e., class four).

## 4. Discussion

This study elicited the preferences of community-dwelling older adults in terms of the “money-following-users” and found out their WTP for greater flexibility and support in decision making with DCE, which is the first study of this topic in the East Asian regions based on our knowledge. DCE has several advantages in measuring stated preference. It gives estimates of preference by simulating the choice behaviours of people in decision making, instead of directly asking them to indicate their preference based on their experience, which reduced biases in measurement. It can also measure the preference for goods/services which do not exist in reality. Although it does not record real choice behaviours in choosing services, the validity of DCE estimates was found to be better than that of experiments and direct surveys of preference [26].

The findings from this study suggest the community-dwelling older adults were willing to pay a higher price for greater flexibility in services in LTC than other attributes, as pre-determined service packages might not be able to fulfil this need. It emphasized the importance of enabling older persons to gain greater flexibility in their own LTC arrangement [27]. The findings are similar to the results reported by a previous study by Consumer-Directed Care in Australia [15] that found older persons preferred full flexibility in changing care plans. The findings of this study also indicate that the flexibility in choice sought by the older persons covers not only service items but also service providers. In light of this, the private provision of relevant community-based LTC can be promoted in order to provide a wider range of provider options for the older users to choose. However, the Australian study found that older persons did not prefer multiple providers, while the participants of this study were willing to pay a higher value for multiple flexible providers. This difference could be the result of the difference in health status of the study sample, as they were eligible users of public-funded aged care, and the average EQ-5D score in Australia was 0.59 [15], a relatively lower level of quality of life, while around half of the participants in this study did not have any IADL impairment. Nevertheless, a substantial proportion (around 33%) of participants in this study were also willing to pay more for a single fixed provider, which could be because they consider it a burden to deal with more than one provider and prefer to stay in a familiar environment and with familiar people [28].

Apart from the preference for flexible service and provider, the preference for more frequent meetings with case managers and social workers as an information source suggests that the decision making of the older persons highly relies on social workers or case managers, although the WTP values were lower than those for level of flexibility. This is comparable to previous studies on the decision making of the older persons, which found that insufficient ability in processing the information may become a barrier for them to make choices [29], and older persons might have difficulties in identifying a better option in LTC service as they did not have enough understanding of them or have cognitive problem [14,30,31]. Based on the experience of individual budgets in the UK, supports in the planning and management of the budgets for older persons that are continuous and have the ability to adapt to the changes in needs and external setting are required to enable users to take control over their care [32]. Taking the preference for both greater flexibility and supports by social workers into consideration, there should be ways to provide adequate information and sufficient support to enable their decision making while avoiding affecting their flexibility in order to fulfil both preferences simultaneously. Experience from Australian Consumer Directed Care suggested that internet-based information resources related to this programme were not enough to support decision making, and the government should consider ways other than the internet and mobile phone for older persons to access relevant information [33]. Another previous study suggested that the capacity of older users to make different types of decisions can be assessed by case managers to determine the level of support required, and those with sufficient cognitive capacity and willingness to make choices themselves can be trained for making decisions on their own [34]. They can be educated to be familiar with the default option of care in the first few months and then be educated to identify their own care needs and weigh care options with the assistance of care managers and decision-aiding tools. Case managers or social workers can also act as a safeguard for those with diminished capacity for decision making. An open dialogue between the carers, older persons and case managers could help them gain common knowledge of what the older persons need for care, which could be helpful to overcome the differences in opinions in the level of flexibility and support [35]. The effectiveness of these measures and relevant assessment tools can be explored in future studies.

Moreover, divided preference among different socio-demographic and health-related subgroups should be recognized, as it is a reflection of different preferences of subgroups of the population, which should be incorporated into the design of the “money-following-users” approach. From the analysis of unobserved preference heterogeneity, substantial heterogeneity was found in the preference for flexibility in providers and information source (whether to receive information from social worker or experience a trial period). Although its association with socio-demographic or health-related characteristics was not clear, this finding highlighted the importance of taking into account the difference of preference across individuals in the design and provision of user-centred care services and implied the need to offer older persons options in the level of flexibility in LTC for them to gain appropriate level of control and choice over LTC based on their own choice and the assessment of the professionals [34]. This is also supported by recent findings in Australia, in which some care receipts were happy to have whatever was offered to them, while the others were not satisfied with the services as they were not capable of negotiating for a more personalized service [36]. From the interactions between attributes and ADL impairment, it was found that people with impairment in ADL had a greater preference for flexible providers but less preference for flexible care services than those without the impairment. It was not surprise to find older persons with the impairment preferred for less flexibility in services, especially determining the services by themselves, as the decision making in LTC is found often considered as a burden or extra responsibility for those with impairment [37,38]. They might have more confidence in the social workers to decide their services or even have a fixed package. On the other hand, the preference for multiple flexible providers implies that older adults with impairment have a greater preference for multiple providers to meet their needs, and it is important for them to decide the provider or caregivers themselves. It also suggests that physical impairment should not be a reason to deprive them of control and choice in LTC; instead they should be offered adequate supports in their decision making capacity, which is influenced by their conditions.

The study filled in the research gap on preference for greater flexibility, control and choice in LTC services for older persons and can be used to inform the design and implementation of relevant self-directed or consumer-directed LTC programmes. Considering the influence of culture and financial status on the preferences for flexibility in choice, the findings of this study might be generalizable to high-income countries and regions in East Asia with similar cultural views on individual autonomy. There were a few limitations that should be addressed. Firstly, the study sample was not entirely random, as those with moderate or severe cognitive impairment were unable to give consent to the survey or unable to understand the DCE questions. For these older persons, their family caregivers usually act as a surrogate in decision making for LTC arrangement, so their preference for flexibility in choice in LTC can be investigated in future studies. Secondly, there were a few limitations in the design of DCE choice sets. There were concerns regarding whether to incorporate “status quo” option (“opt out”) as one of the alternatives in the DCE. One reason was that “Status quo” is usually used in the studies for examining the preference for new programmes where there is an option for users not to opt in and keep the status quo; however, current service models have already been considered and described by the attributes of this DCE, so there was no need for an additional “status quo” option. There was also a risk that too many older adults would choose the “status quo” due to the complexity of the choice tasks [39,40], leading to limited and biased responses. Under the current design, they only needed to choose the alternative with relative higher preferences. On the other hand, the number of choice tasks for one respondent is one of the important components in the DCE design. If this number is too small, there might be higher risk of measurement error for the preferences of each individual, which could affect the test–retest reliability of the DCE survey. Under the current design, the ideal number for each respondent would be around 12 [41]. However, using the ideal number (i.e., n = 12) of choice tasks might lead to heavy cognitive burden on the respondents, especially if the respondents are older adults who need LTC services [40,41]. Following the design of previous similar DCE studies [42,43,44], six choice tasks were put in the questionnaire for each respondent, which can maintain the measurement error at a tolerable level and would not cause too heavy a cognitive burden on older respondents.

## 5. Conclusions

Older persons have higher WTP for greater flexibility in services and providers while relying heavily on social workers for obtaining information and assistance in decision making. Policy makers can take reference from their preference for the improvement of design and implementation of LTC service schemes adopting the “money-following-users” approach by allocating resource to attributes and levels with higher WTP. The enhancement of flexibility in LTC should be supported by policies and regulations which allow the older participants of relevant programmes to make decisions based on their own preferences or their communication with social workers instead of determining the care arrangement for them, which should be facilitated by a working procedure and guidelines that support the decision-making process of the users. Given the substantial heterogeneity found, options should be offered to users in the service provision for them to decide their preferred level of flexibility in LTC to better reflect their needs and divided preferences. Service providers should also be aware of the different preferences across users with different characteristics in service provision and delivery. Further studies can focus on examining the effectiveness and efficiency of relevant care programmes with evidence from the implementation of service models with different levels of flexibility incorporated.

## Figures and Tables

**Table 1:** The attributes and levels of “money-following-users” for DCE.

Attributes	Levels
Flexibility in service providers	(a) Single fixed provider;
	(b) Multiple flexible providers.
Flexibility in care services/plan	(a) Fixed pre-determined service packages;
	(b) Services determined by social workers based on needs;
	(c) Services chosen by oneself.
Case management (Meeting frequency with social workers)	(a) Every month;
	(b) Every 3 months;
	(c) Every 6 months.
Information source	(a) From social workers or staff;
	(b) From family members or friends;
	(c) From a trial period of a service/scheme.
Monthly out-of-pocket payment *	(a) HKD 185 (=USD 23.7);
	(b) HKD 427 (=USD 54.7);
	(c) HKD 802 (=USD 102.8);
	(d) HKD 1200 (=USD 153.8)

* The values of out-of-pocket payment were set based on the community care service voucher pilot scheme phase II.

**Table 2:** Socio-demographic and health-related characteristics of the survey sample.

Socio-Demographic Characteristics	N	Percentage	Health-Related Characteristics	N	Percentage
**Age (Mean ± SD)**	77.31 ± 7.06		**IADL** ^1^		
**Gender**			No impairment	153	48.1%
Male	75	23.6%	With impairment	165	51.9%
Female	243	76.4%	**ADL** ^1^		
**Marital status**			No impairment	225	70.8%
Married	152	47.8%	With impairment	93	29.3%
Widowed	128	40.3%	**Disease prevalence**		
Divorced	20	6.3%	Hypertension	205	64.7%
Never married	18	5.7%	Musculo-skeletal diseases	160	50.3%
**Educational level**			Eye diseases	135	42.5%
No school	46	14.5%	Diabetes	70	22.0%
Primary school	125	39.3%	Heart diseases	59	18.6%
Secondary school	121	38.1%	Depression	30	9.4%
Post-secondary	25	7.9%	Cerebrovascular accident	28	8.8%
Others	1	0.3%	Cancer	12	3.8%
**Living arrangement**			**Healthcare service utilization**		
Alone	129	41.4%	Inpatient (past 1 year)	84	26.4%
Spouse only	111	35.6%	Emergency room (past 6 months)	79	24.8%
Children only	44	14.1%	Public out-patient (past 6 months)	263	82.7%
Spouse and Children	17	5.5%	Private out-patient (past 6 months)	202	63.5%
Domestic helper and/or others	17	5.5%			
**Carer status**					
No carer	205	64.5%			
Elder carer (carer aged 65+)	60	18.9%			
Young carer (carer aged below 65)	53	16.7%			
**Housing**					
Private housing	210	66.0%			
Public housing	108	34.0%			
**Monthly income (HK$)**					
<5000	226	71.1%			
5000+	92	28.9%			
**Total**	318	100%	**Total**	318	100%

^1^ IADL: Instrumental activity of daily living; ADL: Activity of daily living. Bolded text refers to the characteristics investigated in the survey.

**Table 3:** Generalized multinomial logistic model estimates and willingness to pay.

	Coefficient	95%CI ^1^	WTP ^1^	95%CI
Mean				
**Multiple flexible providers (single fixed provider as reference)**	0.87 *	(0.39, 1.35) **	172.73	(106.51, 238.95)
**Service flexibility (fixed package as reference)**				
Services chosen by oneself	1.14 *	(0.61, 1.68) **	226.22	(149.73, 302.72)
Services chosen by social workers	0.89 *	(0.41, 1.37) **	175.60	(108.82, 242.38)
**Case management (meeting every month as reference)**				
Meeting every 3 month	−0.07	(−0.35, 0.22)	−13.07	(−69.53, 43.38)
Meeting every 6 month	−0.49 *	(−0.92, −0.07) *	−97.63	(−174.99, −20.27)
**Information source (from social workers as reference)**				
From family or friends	−0.58 *	(−0.99, −0.16) *	−114.69	(−184.89, −44.48)
From experience in a trial period	−0.37 *	(−0.73, −0.01) *	−73.66	(−137.97, −9.35)
Monthly out-of-pocket payment (per HKD100)	−0.51 *	(−0.67, −0.33) **	-	-
Standard deviation				
**Multiple flexible providers (single fixed provider as reference)**	2.12 *	(1.31, 2.94) **		
**Service (fixed package as reference)**				
Services chosen by oneself	0.06	(−0.67, 0.78)		
Services chosen by social workers	1.01 *	(0.48, 1.54) **		
**Case management (meeting every month as reference)**				
Meeting every 3 month	0.25	(−0.26, 0.76)		
Meeting every 6 month	0.56 *	(0.02, 1.10) *		
**Information source (from social workers as reference)**				
From family or friends	0.42	(−0.05, 0.89)		
From experience in a trial period	−0.84 *	(−1.33, −0.35) *		
**Monthly out-of-pocket payment (per HKD 100)**	0.48 *	(0.32, 0.64) **		
τ statistic (scale heterogeneity)	−0.68 *	(−1.18, −0.18) *		
Log likelihood	−952.98			
N	318			
Obs	3816			

* *p* < 0.05, ** *p* < 0.01; ^1^. CI: Confidence interval; WTP: Willingness to pay (HKD). Bolded text refers the attributes of the discrete choice experiment.

**Table 4:** Predicted 10 most preferred service models and their willingness to pay.

Rank	Flexibility in Providers	Flexibility in Services	Case Management (Meeting Frequency)	Information Source	WTP ^1^ (Out-of-Pocket Monthly Payment)
1	Multiple providers	Chosen by oneself	Every month	From social workers	HKD 611.27/USD 78.4
2	Multiple providers	Chosen by oneself	Every 3 months	From social workers	HKD 598.20/USD 76.7
3	Multiple providers	Chosen by social workers	Every month	From social workers	HKD 560.65/USD 71.9
4	Multiple providers	Chosen by social workers	Every 3 months	From social workers	HKD 547.58/USD 70.2
5	Multiple providers	Chosen by oneself	Every month	From a trial period	HKD 537.61/USD 68.9
6	Multiple providers	Chosen by oneself	Every 3 months	From a trial period	HKD 524.54/USD 67.2
7	Multiple providers	Chosen by oneself	Every 6 months	From social workers	HKD 513.64/USD 65.9
8	Multiple providers	Chosen by oneself	Every month	From family or friends	HKD 496.58/USD 63.7
9	Multiple providers	Chosen by social workers	Every month	From a trial period	HKD 486.99/USD 62.4
10	Multiple providers	Chosen by oneself	Every 3 months	From family or friends	HKD 483.51/USD 62.0

^1^ The WTP of each attribute and level combination generated from the GMNL model with reference to the least-preferable combinations of attributes (i.e., single fixed provider, fixed pre-determined service packages, meeting social workers every 6 months and getting information from family members or friends).

**Table 5:** Willingness to pay (HKD) in different subgroups of the sample.

Attribute Levels	Coefficient	95%CI ^1^
Mean		
**Multiple flexible providers (single fixed provider as reference)**	0.45 *	(0.14, 0.75)
**Service (fixed package as reference)**		
Services chosen by self	1.05 *	(0.71, 1.39)
Services chosen by social workers	0.63 *	(0.36, 0.90)
**Case management (meeting every month as reference)**		
Meeting every 3 month	−0.06	(−0.28, 0.16)
Meeting every 6 month	−0.38 *	(−0.68, −0.09)
**Information source (information from social workers as reference)**		
Information from family or friends	−0.42 *	(−0.69, −0.14)
Information from trial period	−0.28 *	(−0.52, −0.03)
**Cost (per HKD 100)**	−0.41 *	(−0.49, −0.33)
**Interaction**		
Multiple flexible provider * ADL ^1^ impairment	0.74 *	(0.10, 1.38)
Services chosen by self * ADL ^1^ impairment	−0.65 *	(−1.15, −0.15)
Standard deviation		
**Multiple flexible providers (single fixed provider as reference)**	1.59 *	(1.19, 1.99)
**Service (fixed package as reference)**		
Services chosen by self	0.25	(−0.57, 1.06)
Services chosen by social workers	0.77 *	(0.37, 1.17)
**Case management (meeting every month as reference)**		
Meeting every 3 month	0.29	(−0.26, 0.83)
Meeting every 6 month	0.24	(−1.21, 1.70)
**Information source (information from social workers as reference)**		
Information from family or friends	0.38	(−0.02, 0.77)
Information from trial period	−0.59 *	(−0.97, −0.21)
**Cost (per HKD 100)**	0.42 *	(0.32, 0.51)
Log likelihood	−949.61	
AIC	1935.22	
N	318	
Obs	3816	

* *p* < 0.05; ^1^. CI: Confidence interval; the confidence intervals were calculated using delta method. ADL: Activity of daily living. Bolded text refers the attributes of the discrete choice experiment. Shading refers the interactions terms.

## Data Availability

The data presented in this study are available on request from the corresponding author. The data are not publicly available due to protect the confidentiality and privacy of participants.

## Supplementary Materials

The following are available online at https://www.mdpi.com/article/10.3390/ijerph19020686/s1, Tables S1: Goodness of fit of models with different number of classes. Table S2: Coefficient estimates from latent-class logistic model, Table S3: Willingness to pay in different subgroups of the sample.
