# Supplementary material for: Preference of Older Adults for Flexibility in Service and Providers in Community-Based Social Care: A Discrete Choice Experiment
Source: Int J Environ Res Public Health. 2022 Jan 8;19(2):686. doi: 10.3390/ijerph19020686 (PMC8775504; doi:10.3390/ijerph19020686)
Supplement: Supplementary file 1 [file ijerph-19-00686-s001.zip › ijerph-1499529-supplementary.pdf]

Supplementary material. Sensitivity analysis using latent-class logistic model and subgroup analysis of DCE results

There are different statistical models for analysis of discrete choice experiment (DCE) with different assumptions and purposes. Apart from generalized multinomial logistic (GMNL) model and mixed multinomial logistic model (MIXL) as mentioned in the main text which assume the distribution of coefficients (preference) are continuous across individuals, latent-class logistic (LCL) model provides an alternative assumption that the coefficient distribution could be discrete. Therefore, each of the respondents can be assigned to one of a few latent classes with little intra-class variation of preference and substantial variation across classes. Each latent class is defined to have a specific preference profile (Burton & Rigby, 2009; Thiene, Meyerhoff, & De Salvo, 2012). With these classes, LCL model is able to account for preference heterogeneity as well as serial non-participation (Thiene et al., 2012).

Serial non-participation refers to the phenomenon that respondents repeatedly choose the same type of alternatives across different choice tasks (von Haefen, Massey, & Adamowicz, 2005), e.g. choosing the alternative with the highest/lowest level of a particular attribute. This behavior can arise when the respondents actually prefer this attribute level and deem it as the most important factor that affect their decisions, or when they do not want to participate in the process of trading-off attributes or are overwhelmed by complexity of the choice tasks (Burton & Rigby, 2009; Thiene et al., 2012; von Haefen et al., 2005). In order to examine the sensitivity of findings from this DCE study across statistical methods, results from LCL model are reported below and compared with GMNL model results.

The dependent variable was choice of each choice task and independent variables were attribute levels of the DCE. The selection of optimal number of classes in the model depends on the goodness of fit of the model (i.e. AIC, BIC and CAIC). The AIC, BIC and CAIC of models with different number of classes are listed in Table S1. The one with lower value of the information criteria was considered to have higher goodness of fit, so model with 3 or 4 classes were candidates. To avoid missing information on potential preference heterogeneity and serial non-participation, the model with 4 latent classes was selected. Table S2 shows the result of this 4-class model.

Table S1. Goodness of fit of models with different number of classes

Number of classes	Log likelihood	AIC	BIC	CAIC
2	-982.09	1998.18	2062.14	2079.14
3	-950.39	1952.77	2050.59	2076.59
4	-928.39	1926.79	2058.46	2093.46
5	-920.37	1928.73	2094.26	2138.26
6	-903.56	1913.13	2112.52	2165.52
7	-911.94	1947.89	2181.13	2243.13

Table S2. Coefficient estimates from latent-class logistic model

	Class 1		Class 2		Class 3		Class 4	
	β	95% CI	β	95% CI	β	95% CI	β	95% CI
**Flexibility of provider (single fixed provider as reference)**								
Multiple flexible providers (single fixed provider as reference)	6.23	(-0.77, 13.23)	0.73*	(0.44, 1.01)	-2.71*	(-4.51, -0.91)	2.06	(-0.17, 4.28)
**Flexibility of service (fixed package as reference)**								
Services chosen by self	1.11	(-1.42, 3.64)	1.02*	(0.69, 1.35)	-0.48	(-1.47, 0.51)	1.27*	(0.57, 2.91)
Services chosen by social workers	-0.06	(-1.53, 1.41)	0.62*	(0.35, 0.90)	-0.92	(-1.84, 0.01)	1.74*	(-1.61, -0.42)
**Case management (meeting every month as reference)**								
Meeting every 3 month	5.14	(-2.60, 12.88)	-0.14	(-0.39, 0.11)	0.29	(-0.42, 1.00)	-0.34	(-1.22, 1.75)
Meeting every 6 month	1.84	(-2.06, 5.73)	-0.44*	(-0.76, -0.12)	0.18	(-0.91, 1.27)	0.27	(-1.76, 0.52)
**Information source (from social workers as reference)**								
From family or friends	-1.83	(-3.93, 0.28)	-0.23	(-0.53, 0.07)	-0.74	(-1.60, 0.11)	-0.62	(-1.22, 1.75)
From experience in a trial period	-1.99*	(-3.86, -0.13)	0.01	(-0.27, 0.29)	-1.09	(-2.19, 0.01)	-0.95*	(-1.76, 0.52)
**Monthly out-of-pocket payment (per HK$100)**	0.08	(-0.07, 0.22)	-0.07*	(-0.12, -0.03)	-0.46*	(-0.67, -0.26)	-1.02*	(-1.84, -0.06)
Membership								
Income ≥ HK$5000	0.88	(-0.39, 2.15)	1.20*	(0.26, 2.14)	0.59	(-0.53, 1.71)	Reference	-
ADL[1] impairment	0.32	(-0.82, 1.47)	-1.84*	(-2.73, -0.95)	-1.92*	(-3.09, -0.75)	Reference	-
**Class share**	9.60%		39.30%		20.50%		30.60%	

Note: *P<0.05; ADL: activity of daily living

Subgroup analysis of DCE results

Table S3. Willingness to pay in different subgroups of the sample

	Venue for this survey		Monthly income		Informal carers		Living arrangement	
	Community centre	Home	< HK$5000	HK$5000+	No carers	With carers	Living alone	Not living alone
**Flexibility of provider (single fixed provider as reference)**								
Multiple providers	115.03	203.56	143.50	244.51	158.02	111.19	207.78	103.11
95%CI*	(40.48, 189.58)	(104.4, 302.72)	(64.89, 222.12)	(63.01, 426.00)	(76.82, 239.22)	(-24.13, 246.51)	(92.74, 322.82)	(21.61, 184.61)
**Flexibility of service (fixed pre-determined service as reference)**								
Service by self	239.83	149.49	194.21	329.63	205.62	247.03	263.26	262.31
95%CI	(145.85, 333.8)	(32.47, 266.52)	(112.66, 275.75)	(119.86, 539.40)	(115.59, 295.66)	(117.14, 376.93)	(139.65, 386.87)	(166.25, 358.38)
Service by social worker	113.75	145.04	128.01	292.22	129.26	229.51	194.75	237.36
95%CI	(28.78, 198.72)	(31.38, 258.70)	(57.27, 198.75)	(98.09, 486.34)	(48.60, 209.92)	(120.21, 338.8)	(92.04, 297.46)	(146.29, 328.43)
**Case management (meet every month as reference)**								
Meet every 3 month	-50.99	45.61	-25.62	46.75	-21.43	19.69	-0.82	33.36
95%CI	(-115.62, 13.65)	(-35.46, 126.69)	(-85.57, 34.32)	(-106.65, 200.15)	(-91.65, 48.78)	(-68.93, 108.31)	(-98.56, 96.92)	(-38.63, 105.35)
Meet every 6 month	-131.54	-53.91	-111.02	-20.98	-100.32	-39.80	-132.58	-14.89
95%CI	(-231.24, -31.84)	(-161.23, 53.41)	(-195.51, -26.53)	(-230.03, 188.07)	(-193.75, -6.90)	(-156.85, 77.24)	(-256.16, -9.00)	(-110.19, 80.40)
**Information source (information from social workers as reference)**								
Info from family/friends	-111.83	-76.64	-75.91	-232.88	-110.68	-80.78	-107.16	-73.88
95%CI	(-206.15, -17.51)	(-168.34, 15.06)	(-150.39, -1.42)	(-442.19, -23.58)	(-195.74, -25.61)	(-187.36, 25.8)	(-216.19, 1.86)	(-171.23, 23.48)
Info from trial period	-53.98	-81.55	-55.64	-122.98	-69.35	-68.83	-49.13	-82.51
95%CI	(-132.08, 24.13)	(-168.07, 4.97)	(-124.50, 13.21)	(-293.75, 47.78)	(-152.95, 14.25)	(-160.12, 22.45)	(-149.27, 1.01)	(-167.14, 2.11)

Table S3 (Cont'd). Willingness to pay in different subgroups of the sample

	ADL[†] impairment		IADL[‡] impairment		Physical-related IADL impairment		Mental-related IADL impairment	
	No	Yes	No	Yes	No	Yes	No	Yes
**Flexibility of provider (single fixed provider as reference)**								
Multiple providers	149.69	213.90	109.56	201.56	116.42	202.22	159.87	70.29
95%CI	(69.49, 229.89)	(120.75, 307.06)	(12.93, 206.18)	(117.50, 285.61)	(23.57, 209.27)	(115.49, 288.95)	(83.22, 236.51)	(-37.78, 178.36)
**Flexibility of service (fixed pre-determined service as reference)**								
Service by self	281.84	98.25	221.78	234.36	222.64	252.24	254.69	176.55
95%CI	(191.17, 372.50)	(-8.71, 205.21)	(110.56, 333.01)	(140.3, 328.42)	(116.35, 328.93)	(143.18, 361.30)	(163.35, 346.04)	(48.30, 304.80)
Service by social worker	150.64	108.37	192.80	161.91	150.57	197.15	200.86	147.77
95%CI	(71.44, 229.84)	(16.56, 200.18)	(91.29, 294.32)	(78.47, 245.35)	(56.55, 244.59)	(104.61, 289.70)	(119.84, 281.89)	(37.84, 257.69)
**Case management (meet every month as reference)**								
Meet every 3 month	-20.65	8.87	-16.12	2.17	-29.32	24.15	-38.05	83.35
95%CI	(-87.98, 46.69)	(-79.77, 97.52)	(-103.09, 70.85)	(-67.20, 71.55)	(-105.68, 47.04)	(-54.8, 103.10)	(-106.67, 30.58)	(-5.99, 172.69)
Meet every 6 month	-57.98	-76.98	-133.84	-52.52	-140.23	-39.38	-140.30	-19.24
95%CI	(-156.80, 40.84)	(-176.82, 22.86)	(-248.96, -18.72)	(-151.35, 46.31)	(-247.96, -32.49)	(-147.60, 68.83)	(-238.51, -42.10)	(-127.77, 89.29)
**Information source (information from social workers as reference)**								
Info from family/friends	-131.02	-62.39	-167.50	-23.72	-163.38	-56.15	-126.85	-68.78
95%CI	(-218.96, -43.08)	(-153.95, 29.17)	(-283.38, -51.63)	(-104.74, 57.29)	(-266.79, -59.98)	(-148.61, 36.32)	(-214.47, -39.22)	(-157.9, 20.35)
Info from trial period	-71.22	-92.32	-123.29	15.71	-120.74	-15.13	-66.54	-51.04
95%CI	(-143.35, 0.90)	(-184.32, -0.32)	(-220.82, -25.77)	(-61.06, 92.47)	(-210.13, -31.35)	(-103.38, 73.12)	(-142.94, 9.87)	(-152.35, 50.26)

Note: *CI: confidence interval; The confidence intervals were calculated using Delta method. †ADL: Activities of Daily Living; ‡ IADL: Instrumental Activities of Daily Living

Reference

Burton, M., & Rigby, D. (2009). Hurdle and latent class approaches to serial non-participation in choice models. *Environmental and Resource Economics, 42*(2), 211.

Kaambwa, B., Lancsar, E., McCaffrey, N., Chen, G., Gill, L., Cameron, I. D., . . . Ratcliffe, J. (2015). Investigating consumers' and informal carers' views and preferences for consumer directed care: A discrete choice experiment. *Social Science & Medicine, 140*, 81-94.

Thiene, M., Meyerhoff, J., & De Salvo, M. (2012). Scale and taste heterogeneity for forest biodiversity: Models of serial nonparticipation and their effects. *Journal of forest economics, 18*(4), 355-369.

von Haefen, R. H., Massey, D. M., & Adamowicz, W. L. (2005). Serial nonparticipation in repeated discrete choice models. *American Journal of Agricultural Economics, 87*(4), 1061-1076.

Questionnaire for preference of older adults for community-based long-term care delivery mode

(Original version is in Chinese version)

**Section 1. Preference for care mode**	**Start time:________________**	**End time ________________**
**DCE version A-F**	**A ☐ B ☐ C ☐ D ☐ E ☐ F ☐**	
1. Service mode 1.1 ☐	Service mode1.2 ☐	
2. Service mode2.1 ☐	Service mode2.2 ☐	
3. Service mode3.1 ☐	Service mode3.2 ☐	
4. Service mode4.1 ☐	Service mode4.2 ☐	
5. Service mode5.1 ☐	Service mode5.2 ☐	
6. Service mode6.1 ☐	Service mode6.2 ☐	
**Section 2: Health status and functions**		
Instrumental Activities of Daily Living (in the past month)		
1. Shopping……………………….	☐0 Completely unable to shop<br>☐1 Needs to be accompanied on any shopping trip<br>☐2 Shops independently for small purchases<br>☐3 Takes care of all shopping needs independently	
2. Mode of Transportation………….	☐0 Does not travel at all<br>☐1 Travel limited to taxi or automobile with assistance of another<br>☐2 Travels on public transportation when assisted or accompanied by another<br>☐3 Arranges own travel via taxi, but does not otherwise use public transportation<br>☐4 Travels independently on public transportation or drives own car	
3. Food Preparation …………………..	☐0 Needs to have meals prepared and served<br>☐1 Heats and serves prepared meals or prepares meals but does not maintain adequate diet<br>☐2 Prepares adequate meals if supplied with ingredients<br>☐3 Plans, prepares, and serves adequate meals independently	
4. Housekeeping ……………………..	☐0 Does not participate in any housekeeping tasks<br>☐1 Needs help with all home maintenance tasks<br>☐2 Performs light daily tasks, but cannot maintain acceptable level of cleanliness<br>☐3 Performs light daily tasks such as dishwashing, bed making<br>☐4 Maintains house alone with occasion assistance (heavy work)	
5. Laundry …………………………….	☐0 All laundry must be done by others<br>☐1 Launders small items, rinses socks, stockings, etc<br>☐2 Does personal laundry completely	
6. Ability to Use Telephone ……..	☐0 Does not use telephone at all<br>☐1 Answers telephone, but does not dial<br>☐2 Dials a few well-known numbers	

		☐[3] Operates telephone on own initiative; looks up and dials numbers
7.	Responsibility for Own Medications	☐[0] Is not capable of dispensing own medication<br>☐[1] Takes responsibility if medication is prepared in advance in separate dosages<br>☐[2] Is responsible for taking medication in correct dosages at correct time
8.	Ability to Handle Finances ……….	☐[0] Incapable of handling money<br>☐[1] Manages day-to-day purchases, but needs help with banking, major purchases, etc<br>☐[2] Manages financial matters independently (budgets, writes checks, pays rent and bills, goes to bank); collects and keeps track of income
**Activities of Daily Living (in the past month)**		
9.	Feeding ………………………………	☐[0] unable<br>☐[1] needs help cutting, spreading butter, etc., or requires modified diet<br>☐[2] independent
10.	Bathing ………………………………	☐[0] dependent<br>☐[1] independent (or in shower)
11.	Grooming ………………………..	☐[0] needs to help with personal care<br>☐[1] independent face/hair/teeth/shaving (implements provided)
12.	Dressing ………………………..	☐[0] dependent<br>☐[1] needs help but can do about half unaided<br>☐[2] independent (including buttons, zips, laces, etc.)
13.	Bowels ………………………..	☐[0] incontinent (or needs to be given enemas)<br>☐[1] occasional accident<br>☐[2] continent
14.	Bladder ………………………..	☐[0] incontinent, or catheterized and unable to manage alone<br>☐[1] occasional accident<br>☐[2] continent
15.	Toilet use ………………………………	☐[0] dependent<br>☐[1] needs some help, but can do something alone<br>☐[2] independent (on and off, dressing, wiping)
16.	Transfer …………………………….	☐[0] unable, no sitting balance<br>☐[1] major help (one or two people, physical), can sit<br>☐[2] minor help (verbal or physical)<br>☐[3] independent
17.	Mobility ………………………………	☐[0] immobile or < 50 yards<br>☐[1] wheelchair independent, including corners, > 50 metres<br>☐[2] walks with help of one person (verbal or physical) > 50 metres<br>☐[3] independent (but may use any aid; for example, stick) > 50 metres
18.	Stairs ………………………..	☐[0] unable<br>☐[1] needs help (verbal, physical, carrying aid)<br>☐[2] independent

Background information

**Section 3: Personal information**				
19. Sex……………………………….	☐^{0} Male	☐^{1} Female		
20. Birth date……………………….	(DD/MM/YYYY)			
21. Marital status……………………….	☐^{0} Married	☐^{1} Widowed	☐^{2} Divorce/separate	☐^{3} Single
22. Living status……………………….	☐^{0}Alone	☐^{1} Spouse only	☐^{2} Children ☐^{3} Helper	☐^{4}Other
23. Education level……………………….	☐^{0}No schooling	☐^{1}Primary school	☐^{2} Secondary school	☐^{3} High school
	☐^{4} Pre-course	☐^{5} University or above	☐^{6} Other	
24. Monthly income（HK$）………	☐^{0} <1,000	☐^{1} 1,000-2,999	☐^{2} 3,000-4,999	
	☐^{3} 5,000-9,999	☐^{4} 10,000-19,999	☐^{5} >20,000	
**Section 4: Housing status**				
25. Tentative…………………….	☐^{0}Rent	☐^{1}Own		
26. Type of housing				
29.1 Private…………… ☐	☐^{0}Independent unit	☐^{1}Sub-divided unit	☐^{2}Others	
29.2 Public ………… ☐	☐^{0} Independent unit		☐^{1}Home for aged	
	☐^{2} Other			
**Section 5: Caregiver status**				
27. Have an informal caregiver or not……………………	☐^{0}No ->Jump to Q31			
	☐^{1}Yes ---------------->	31.1 Age of caregiver:	☐^{0} <65yrs	☐^{1} ≥65yrs
28. Relationship with carer…………..	☐^{0}Spouse	☐^{1}Children	☐^{2}Other relative	
	☐^{3}Friends/neighbor	☐^{4}Domestic helper	☐^{5}Other	
29. Proximity………..	☐^{0}Living together	☐^{1}Same buiding	☐^{2} Same estate	
	☐^{3}Nearby estate	☐^{4}Same district	☐^{5}None of above	
30. Type of care (multiple choices)	☐^{0}Emotional support			
	☐^{1}IADL care			
	☐^{2}ADL care			
**Section 6: Health status**				
31. Have you ever been diagnosed to have following conditions：				

a. Hypertension………	☐^{0}No	☐^{1}Yes	e. Cataract/ Glaucoma	☐^{0}No	☐^{1}Yes
b. Diabetes……………..	☐^{0}No	☐^{1}Yes	f. Stroke	☐^{0}No	☐^{1}Yes
c. Cancer………………..	☐^{0}No	☐^{1}Yes	g. Depression	☐^{0}No	☐^{1}Yes
d. Heart disease …	☐^{0}No	☐^{1}Yes	h. Musculoskeletal disease	☐^{0}No	☐^{1}Yes

32. Frequency of pain	☐^{0} No pain	☐^{1} Less than once a day	☐^{2} Once or multiple times a day	☐^{3} Persistent pain

Choice Sets for Discrete Choice Experiment

Hypothetical questions

Assuming that you have been offered these different alternatives for arrangement of delivering community-based long-term care services as described in the six choice questions below. Please read and compare the two alternatives in each choice questions, and select a more attractive one based on your preference. There is no right or wrong for your answers.

Choice Experiment Questionnaire - - Block A

1.

	Service Mode1		Service Mode2
Flexibility of providers	Single fixed provider		Multiple flexible provider
Flexibility of care services/plan	Services determined by social workers based on needs		Services chosen by oneself
Monthly out-of-pocket payment	HKD185		HKD427
Meeting frequency with social workers	Every month		Every 3 months
Information source	From social workers or staff		From a trial period of the service/scheme

Which one do you prefer: Service Mode1 □ ; Service Mode2 □

2.

	Service Mode1		Service Mode2
Flexibility of providers	Single fixed provider		Multiple flexible provider
Flexibility of care services/plan	Services chosen by oneself		Fixed pre-determined service packages
Monthly out-of-pocket payment	HKD185		HKD427
Meeting frequency with social workers	Every 3 months		Every month
Information source	From social workers or staff		From a trial period of the service/scheme

Which one do you prefer: Service Mode1 □ ; Service Mode2 □

3.

	Service Mode1		Service Mode2
Flexibility of providers	Single fixed provider		Multiple flexible provider
Flexibility of care services/plan	Services determined by social workers based on needs		Fixed pre-determined service packages
Monthly out-of-pocket payment	HKD802		HKD1200
Meeting frequency with social workers	Every month		Every 3 months
Information source	From a trial period of the service/scheme		From family members or friends

Which one do you prefer: Service Mode1 □ ; Service Mode2 □

4.

	Service Mode1		Service Mode2
Flexibility of providers	Multiple flexible provider		Single fixed provider
Flexibility of care services/plan	Fixed pre-determined service packages		Fixed pre-determined service packages
Monthly out-of-pocket payment	HKD185		HKD1200
Meeting frequency with social workers	Every 3 months		Every 6 months
Information source	From a trial period of the service/scheme		From family members or friends

Which one do you prefer: Service Mode1 □ ; Service Mode2 □

5.

	Service Mode1		Service Mode2
Flexibility of providers	Single fixed provider		Multiple flexible provider
Flexibility of care services/plan	Services chosen by oneself		Services determined by social workers based on needs
Monthly out-of-pocket payment	HKD427		HKD185
Meeting frequency with social workers	Every month		Every 3 months
Information source	From a trial period of the service/scheme		From social workers or staff

Which one do you prefer: Service Mode1 □ ; Service Mode2 □

6.

	Service Mode1		Service Mode2
Flexibility of providers	Multiple flexible provider		Single fixed provider
Flexibility of care services/plan	Services chosen by oneself		Fixed pre-determined service packages
Monthly out-of-pocket payment	HKD185		HKD427
Meeting frequency with social workers	Every 6 months		Every month
Information source	From a trial period of the service/scheme		From family members or friends

Which one do you prefer: Service Mode1 □ ; Service Mode2 □

Choice Experiment Questionnaire - - Block B

1.

	Service Mode1		Service Mode2
Flexibility of providers	Single fixed provider		Multiple flexible provider
Flexibility of care services/plan	Services chosen by oneself		Services determined by social workers based on needs
Monthly out-of-pocket payment	HKD802		HKD1200
Meeting frequency with social workers	Every month		Every 6 months
Information source	From social workers or staff		From a trial period of the service/scheme

Which one do you prefer: Service Mode1 □ ; Service Mode2 □

2.

	Service Mode1		Service Mode2
Flexibility of providers	Single fixed provider		Multiple flexible provider
Flexibility of care services/plan	Fixed pre-determined service packages		Services determined by social workers based on needs
Monthly out-of-pocket payment	HKD427		HKD802
Meeting frequency with social workers	Every 3 months		Every month
Information source	From a trial period of the service/scheme		From family members or friends

Which one do you prefer: Service Mode1 □ ; Service Mode2 □

3.

	Service Mode1		Service Mode2
Flexibility of providers	Single fixed provider		Single fixed provider
Flexibility of care services/plan	Fixed pre-determined service packages		Services determined by social workers based on needs
Monthly out-of-pocket payment	HKD185		HKD802
Meeting frequency with social workers	Every month		Every 3 months
Information source	From a trial period of the service/scheme		From family members or friends

Which one do you prefer: Service Mode1 □ ; Service Mode2 □

4.

	Service Mode1		Service Mode2
Flexibility of providers	Single fixed provider		Multiple flexible provider
Flexibility of care services/plan	Services chosen by oneself		Fixed pre-determined service packages
Monthly out-of-pocket payment	HKD427		HKD802
Meeting frequency with social workers	Every month		Every 6 months
Information source	From social workers or staff		From family members or friends

Which one do you prefer: Service Mode1 □ ; Service Mode2 □

5.

	Service Mode1		Service Mode2
Flexibility of providers	Single fixed provider		Multiple flexible provider
Flexibility of care services/plan	Services determined by social workers based on needs		Fixed pre-determined service packages
Monthly out-of-pocket payment	HKD185		HKD802
Meeting frequency with social workers	Every month		Every 3 months
Information source	From family members or friends		From social workers or staff

Which one do you prefer: Service Mode1 □ ; Service Mode2 □

6.

	Service Mode1		Service Mode2
Flexibility of providers	Multiple flexible provider		Single fixed provider
Flexibility of care services/plan	Services determined by social workers based on needs		Fixed pre-determined service packages
Monthly out-of-pocket payment	HKD427		HKD185
Meeting frequency with social workers	Every month		Every 3 months
Information source	From a trial period of the service/scheme		From family members or friends

Which one do you prefer: Service Mode1 □ ; Service Mode2 □

Choice Experiment Questionnaire - - Block C

1.

	Service Mode1		Service Mode2
Flexibility of providers	Multiple flexible provider		Single fixed provider
Flexibility of care services/plan	Services chosen by oneself		Services determined by social workers based on needs
Monthly out-of-pocket payment	HKD802		HKD427
Meeting frequency with social workers	Every 3 months		Every 6 months
Information source	From family members or friends		From a trial period of the service/scheme

Which one do you prefer: Service Mode1 □ ; Service Mode2 □

2.

	Service Mode1		Service Mode2
Flexibility of providers	Single fixed provider		Multiple flexible provider
Flexibility of care services/plan	Services determined by social workers based on needs		Fixed pre-determined service packages
Monthly out-of-pocket payment	HKD427		HKD1200
Meeting frequency with social workers	Every 3 months		Every 6 months
Information source	From social workers or staff		From family members or friends

Which one do you prefer: Service Mode1 □ ; Service Mode2 □

3.

	Service Mode1		Service Mode2
Flexibility of providers	Multiple flexible provider		Single fixed provider
Flexibility of care services/plan	Services chosen by oneself		Services determined by social workers based on needs
Monthly out-of-pocket payment	HKD802		HKD427
Meeting frequency with social workers	Every month		Every 3 months
Information source	From a trial period of the service/scheme		From family members or friends

Which one do you prefer: Service Mode1 □ ; Service Mode2 □

4.

	Service Mode1		Service Mode2
Flexibility of providers	Single fixed provider		Multiple flexible provider
Flexibility of care services/plan	Services determined by social workers based on needs		Services chosen by oneself
Monthly out-of-pocket payment	HKD185		HKD427
Meeting frequency with social workers	Every 6 months		Every 3 months
Information source	From a trial period of the service/scheme		From social workers or staff

Which one do you prefer: Service Mode1 □ ; Service Mode2 □

5.

	Service Mode1		Service Mode2
Flexibility of providers	Multiple flexible provider		Single fixed provider
Flexibility of care services/plan	Services determined by social workers based on needs		Services chosen by oneself
Monthly out-of-pocket payment	HKD1200		HKD427
Meeting frequency with social workers	Every month		Every 3 months
Information source	From a trial period of the service/scheme		From family members or friends

Which one do you prefer: Service Mode1 □ ; Service Mode2 □

6.

	Service Mode1		Service Mode2
Flexibility of providers	Multiple flexible provider		Single fixed provider
Flexibility of care services/plan	Services determined by social workers based on needs		Services chosen by oneself
Monthly out-of-pocket payment	HKD802		HKD185
Meeting frequency with social workers	Every 6 months		Every 3 months
Information source	From social workers or staff		From a trial period of the service/scheme

Which one do you prefer: Service Mode1 □ ; Service Mode2 □

Choice Experiment Questionnaire - - Block D

1.

	Service Mode1		Service Mode2
Flexibility of providers	Multiple flexible provider		Single fixed provider
Flexibility of care services/plan	Fixed pre-determined service packages		Services determined by social workers based on needs
Monthly out-of-pocket payment	HKD427		HKD802
Meeting frequency with social workers	Every 6 months		Every month
Information source	From family members or friends		From a trial period of the service/scheme

Which one do you prefer: Service Mode1 □ ; Service Mode2 □

2.

	Service Mode1		Service Mode2
Flexibility of providers	Multiple flexible provider		Single fixed provider
Flexibility of care services/plan	Fixed pre-determined service packages		Services determined by social workers based on needs
Monthly out-of-pocket payment	HKD427		HKD1200
Meeting frequency with social workers	Every 3 months		Every month
Information source	From a trial period of the service/scheme		From social workers or staff

Which one do you prefer: Service Mode1 □ ; Service Mode2 □

3.

	Service Mode1		Service Mode2
Flexibility of providers	Multiple flexible provider		Single fixed provider
Flexibility of care services/plan	Fixed pre-determined service packages		Services determined by social workers based on needs
Monthly out-of-pocket payment	HKD1200		HKD427
Meeting frequency with social workers	Every 3 months		Every 6 months
Information source	From a trial period of the service/scheme		From family members or friends

Which one do you prefer: Service Mode1 □ ; Service Mode2 □

4.

	Service Mode1		Service Mode2
Flexibility of providers	Multiple flexible provider		Single fixed provider
Flexibility of care services/plan	Services chosen by oneself		Fixed pre-determined service packages
Monthly out-of-pocket payment	HKD1200		HKD802
Meeting frequency with social workers	Every month		Every 3 months
Information source	From a trial period of the service/scheme		From social workers or staff

Which one do you prefer: Service Mode1 □ ; Service Mode2 □

5.

	Service Mode1		Service Mode2
Flexibility of providers	Single fixed provider		Multiple flexible provider
Flexibility of care services/plan	Services chosen by oneself		Fixed pre-determined service packages
Monthly out-of-pocket payment	HKD1200		HKD802
Meeting frequency with social workers	Every 3 months		Every month
Information source	From a trial period of the service/scheme		From social workers or staff

Which one do you prefer: Service Mode1 □ ; Service Mode2 □

6.

	Service Mode1		Service Mode2
Flexibility of providers	Multiple flexible provider		Single fixed provider
Flexibility of care services/plan	Services determined by social workers based on needs		Services chosen by oneself
Monthly out-of-pocket payment	HKD1200		HKD802
Meeting frequency with social workers	Every month		Every 6 months
Information source	From family members or friends		From a trial period of the service/scheme

Which one do you prefer: Service Mode1 □ ; Service Mode2 □

Choice Experiment Questionnaire - - Block E

1.

	Service Mode1		Service Mode2
Flexibility of providers	Multiple flexible provider		Single fixed provider
Flexibility of care services/plan	Services determined by social workers based on needs		Fixed pre-determined service packages
Monthly out-of-pocket payment	HKD802		HKD185
Meeting frequency with social workers	Every 3 months		Every month
Information source	From a trial period of the service/scheme		From family members or friends

Which one do you prefer: Service Mode1 □ ; Service Mode2 □

2.

	Service Mode1		Service Mode2
Flexibility of providers	Single fixed provider		Multiple flexible provider
Flexibility of care services/plan	Fixed pre-determined service packages		Services chosen by oneself
Monthly out-of-pocket payment	HKD802		HKD185
Meeting frequency with social workers	Every 3 months		Every month
Information source	From a trial period of the service/scheme		From family members or friends

Which one do you prefer: Service Mode1 □ ; Service Mode2 □

3.

	Service Mode1		Service Mode2
Flexibility of providers	Multiple flexible provider		Single fixed provider
Flexibility of care services/plan	Services chosen by oneself		Services determined by social workers based on needs
Monthly out-of-pocket payment	HKD802		HKD1200
Meeting frequency with social workers	Every 6 months		Every 3 months
Information source	From social workers or staff		From a trial period of the service/scheme

Which one do you prefer: Service Mode1 □ ; Service Mode2 □

4.

	Service Mode1		Service Mode2
Flexibility of providers	Multiple flexible provider		Single fixed provider
Flexibility of care services/plan	Services determined by social workers based on needs		Fixed pre-determined service packages
Monthly out-of-pocket payment	HKD185		HKD802
Meeting frequency with social workers	Every 3 months		Every month
Information source	From a trial period of the service/scheme		From family members or friends

Which one do you prefer: Service Mode1 □ ; Service Mode2 □

5.

	Service Mode1		Service Mode2
Flexibility of providers	Single fixed provider		Multiple flexible provider
Flexibility of care services/plan	Fixed pre-determined service packages		Services determined by social workers based on needs
Monthly out-of-pocket payment	HKD802		HKD427
Meeting frequency with social workers	Every month		Every 3 months
Information source	From a trial period of the service/scheme		From family members or friends

Which one do you prefer: Service Mode1 □ ; Service Mode2 □

6.

	Service Mode1		Service Mode2
Flexibility of providers	Multiple flexible provider		Multiple flexible provider
Flexibility of care services/plan	Services chosen by oneself		Services determined by social workers based on needs
Monthly out-of-pocket payment	HKD1200		HKD802
Meeting frequency with social workers	Every 6 months		Every 3 months
Information source	From social workers or staff		From family members or friends

Which one do you prefer: Service Mode1 □ ; Service Mode2 □

Choice Experiment Questionnaire - - Block F

1.

	Service Mode1		Service Mode2
Flexibility of providers	Single fixed provider		Multiple flexible provider
Flexibility of care services/plan	Services chosen by oneself		Fixed pre-determined service packages
Monthly out-of-pocket payment	HKD1200		HKD185
Meeting frequency with social workers	Every 3 months		Every 6 months
Information source	From family members or friends		From a trial period of the service/scheme

Which one do you prefer: Service Mode1 □ ; Service Mode2 □

2.

	Service Mode1		Service Mode2
Flexibility of providers	Single fixed provider		Multiple flexible provider
Flexibility of care services/plan	Services chosen by oneself		Fixed pre-determined service packages
Monthly out-of-pocket payment	HKD802		HKD185
Meeting frequency with social workers	Every 6 months		Every 3 months
Information source	From family members or friends		From social workers or staff

Which one do you prefer: Service Mode1 □ ; Service Mode2 □

3.

	Service Mode1		Service Mode2
Flexibility of providers	Multiple flexible provider		Single fixed provider
Flexibility of care services/plan	Services determined by social workers based on needs		Fixed pre-determined service packages
Monthly out-of-pocket payment	HKD427		HKD1200
Meeting frequency with social workers	Every month		Every 3 months
Information source	From social workers or staff		From a trial period of the service/scheme

Which one do you prefer: Service Mode1 □ ; Service Mode2 □

4.

	Service Mode1		Service Mode2
Flexibility of providers	Multiple flexible provider		Single fixed provider
Flexibility of care services/plan	Fixed pre-determined service packages		Services determined by social workers based on needs
Monthly out-of-pocket payment	HKD185		HKD802
Meeting frequency with social workers	Every month		Every 6 months
Information source	From family members or friends		From social workers or staff

Which one do you prefer: Service Mode1 □ ; Service Mode2 □

5.

	Service Mode1		Service Mode2
Flexibility of providers	Single fixed provider		Multiple flexible provider
Flexibility of care services/plan	Services chosen by oneself		Services determined by social workers based on needs
Monthly out-of-pocket payment	HKD427		HKD1200
Meeting frequency with social workers	Every 6 months		Every 3 months
Information source	From family members or friends		From social workers or staff

Which one do you prefer: Service Mode1 □ ; Service Mode2 □

6.

	Service Mode1		Service Mode2
Flexibility of providers	Multiple flexible provider		Single fixed provider
Flexibility of care services/plan	Fixed pre-determined service packages		Services determined by social workers based on needs
Monthly out-of-pocket payment	HKD427		HKD802
Meeting frequency with social workers	Every month		Every 3 months
Information source	From social workers or staff		From family members or friends

Which one do you prefer: Service Mode1 □ ; Service Mode2 □
